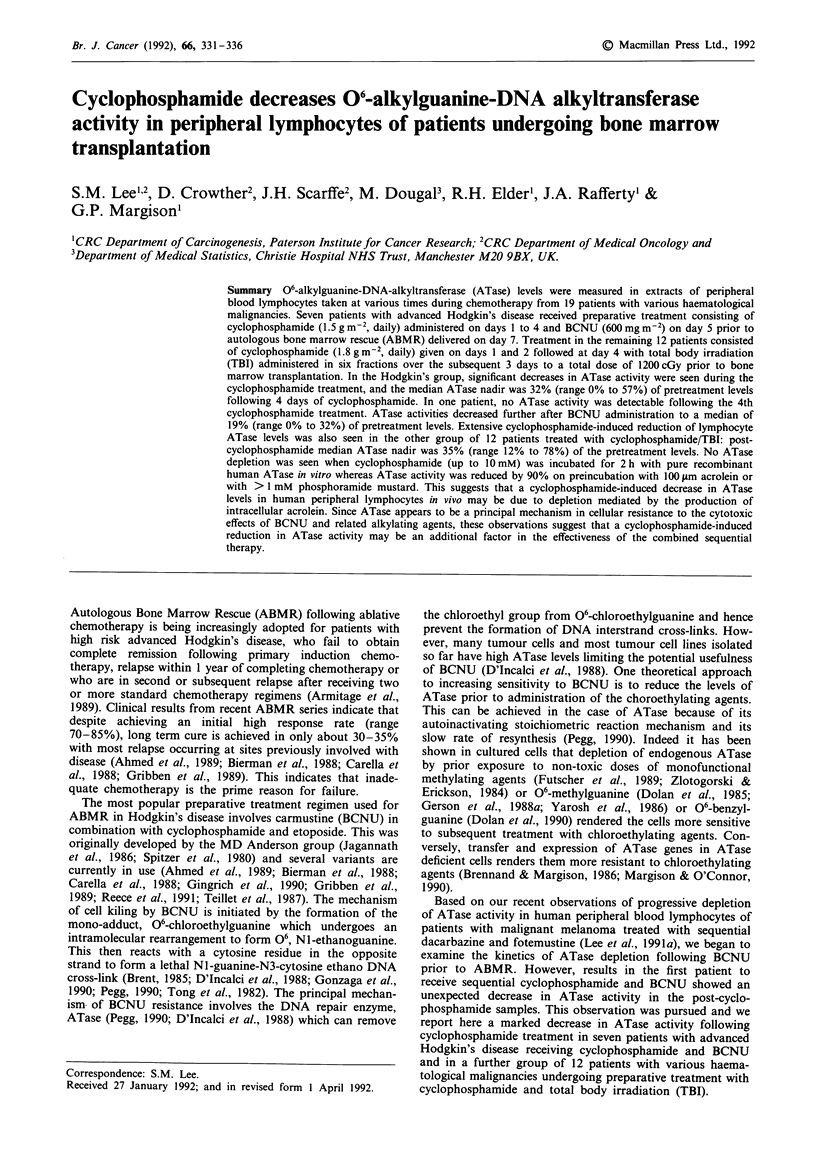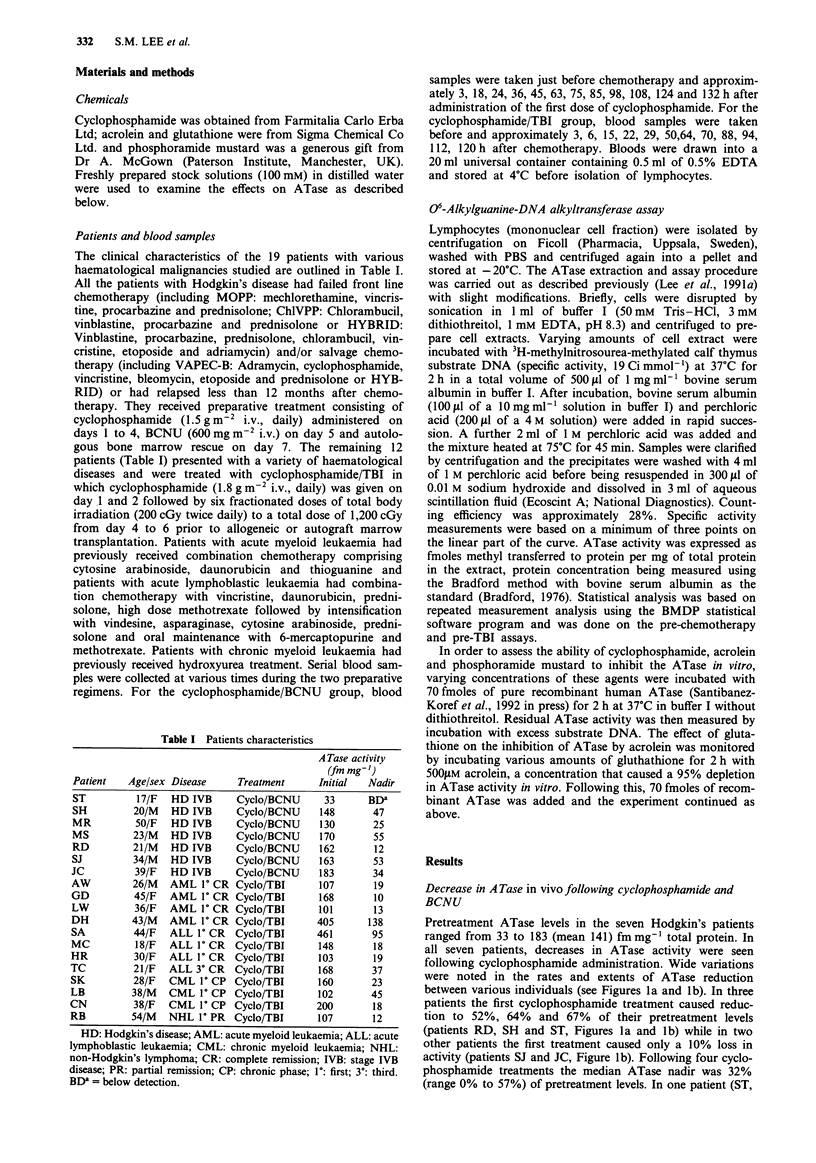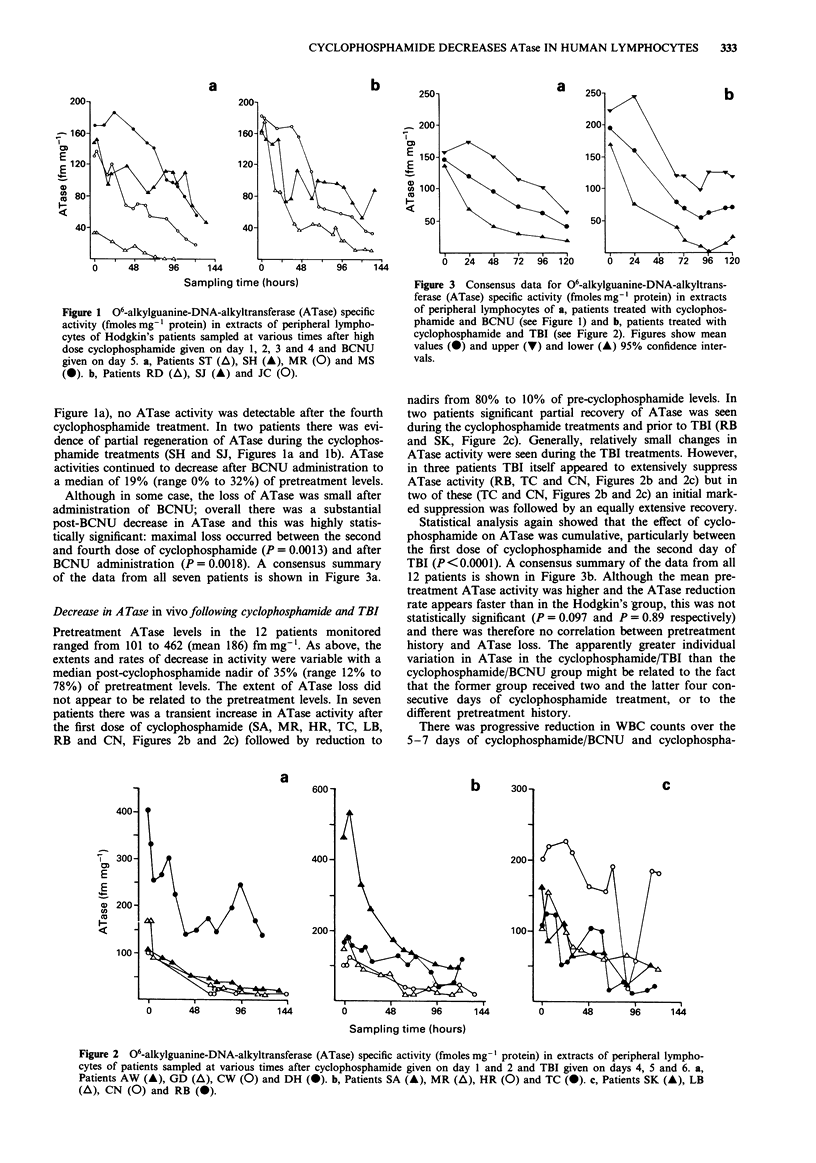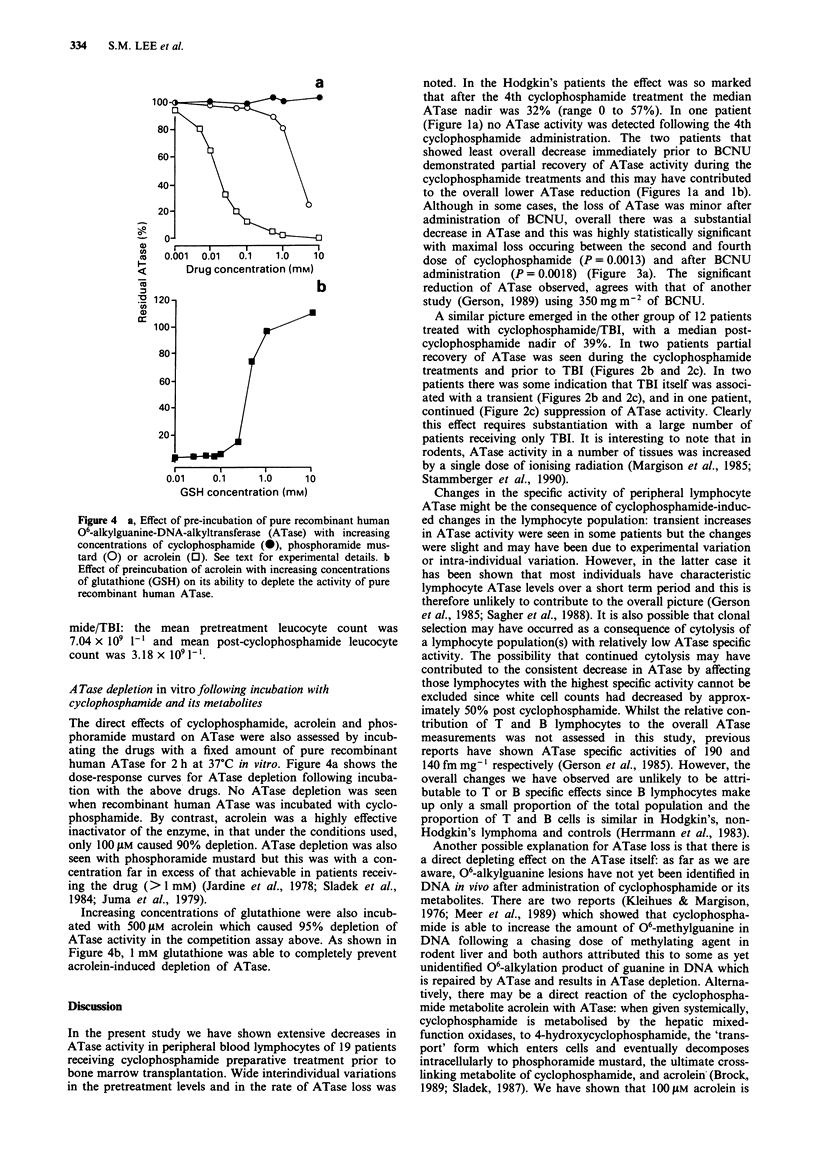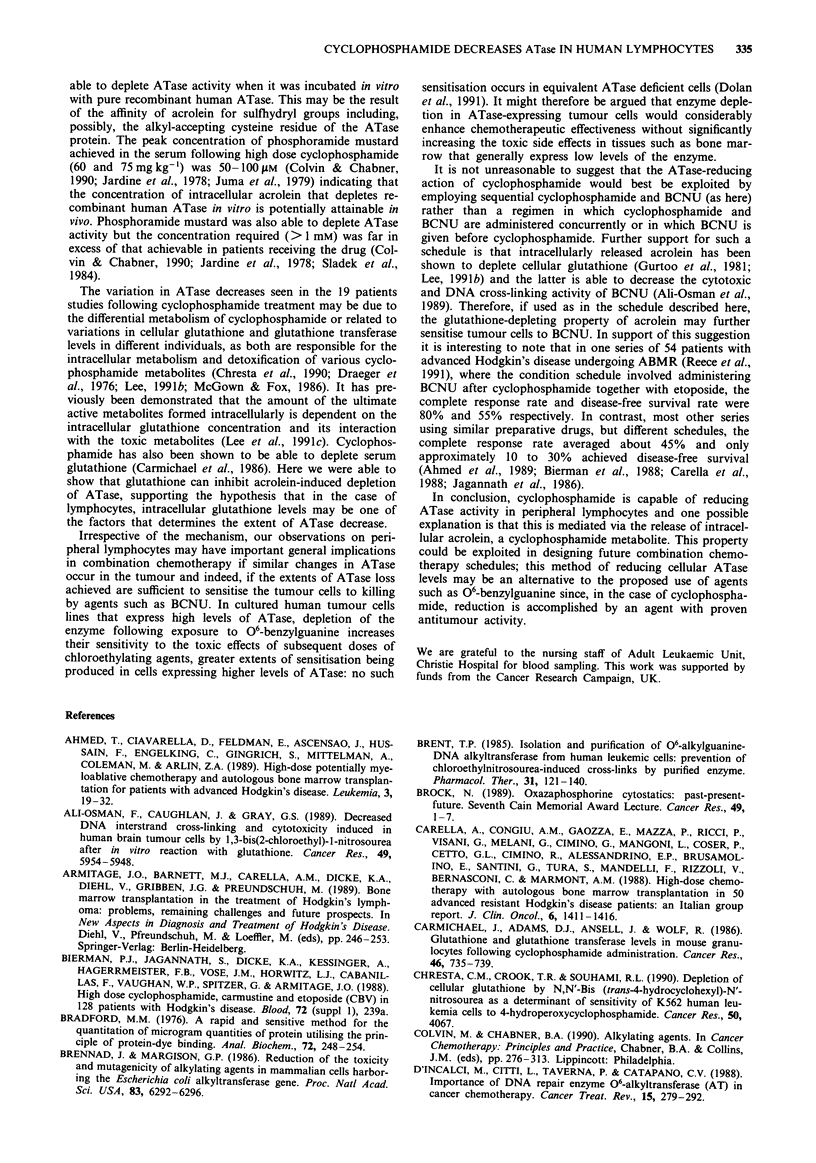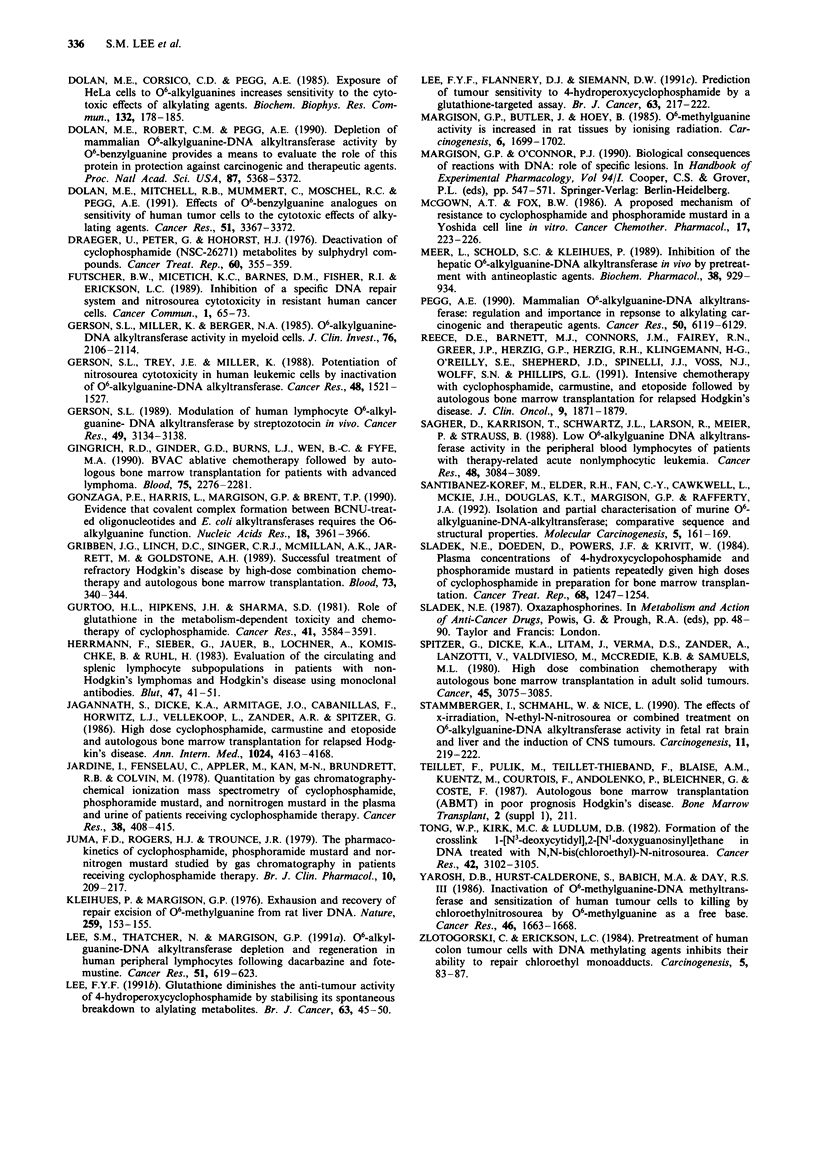# Cyclophosphamide decreases O6-alkylguanine-DNA alkyltransferase activity in peripheral lymphocytes of patients undergoing bone marrow transplantation.

**DOI:** 10.1038/bjc.1992.265

**Published:** 1992-08

**Authors:** S. M. Lee, D. Crowther, J. H. Scarffe, M. Dougal, R. H. Elder, J. A. Rafferty, G. P. Margison

**Affiliations:** CRC Department of Carcinogenesis, Paterson Institute for Cancer Research, Manchester, UK.

## Abstract

O6-alkylguanine-DNA-alkyltransferase (ATase) levels were measured in extracts of peripheral blood lymphocytes taken at various times during chemotherapy from 19 patients with various haematological malignancies. Seven patients with advanced Hodgkin's disease received preparative treatment consisting of cyclophosphamide (1.5 g m-2, daily) administered on days 1 to 4 and BCNU (600 mg m-2) on day 5 prior to autologous bone marrow rescue (ABMR) delivered on day 7. Treatment in the remaining 12 patients consisted of cyclophosphamide (1.8 g m-2, daily) given on days 1 and 2 followed at day 4 with total body irradiation (TBI) administered in six fractions over the subsequent 3 days to a total dose of 1200 cGy prior to bone marrow transplantation. In the Hodgkin's group, significant decreases in ATase activity were seen during the cyclophosphamide treatment, and the median ATase nadir was 32% (range 0% to 57%) of pretreatment levels following 4 days of cyclophosphamide. In one patient, no ATase activity was detectable following the 4th cyclophosphamide treatment. ATase activities decreased further after BCNU administration to a median of 19% (range 0% to 32%) of pretreatment levels. Extensive cyclophosphamide-induced reduction of lymphocyte ATase levels was also seen in the other group of 12 patients treated with cyclophosphamide/TBI: postcyclophosphamide median ATase nadir was 35% (range 12% to 78%) of the pretreatment levels. No ATase depletion was seen when cyclophosphamide (up to 10 mM) was incubated for 2 h with pure recombinant human ATase in vitro whereas ATase activity was reduced by 90% on preincubation with 100 microns acrolein or with greater than 1 mM phosphoramide mustard. This suggests that a cyclophosphamide-induced decrease in ATase levels in human peripheral lymphocytes in vivo may be due to depletion mediated by the production of intracellular acrolein. Since ATase appears to be a principal mechanism in cellular resistance to the cytotoxic effects of BCNU and related alkylating agents, these observations suggest that a cyclophosphamide-induced reduction in ATase activity may be an additional factor in the effectiveness of the combined sequential therapy.


Br. J. Cancer (1992), 66, 331-336 © Macmillan Press Ltd., 1992

### Cyclophosphamide decreases $O^6$-alkylguanine-DNA alkyltransferase activity in peripheral lymphocytes of patients undergoing bone marrow transplantation

S.M. Lee[1,2], D. Crowther[2], J.H. Scarffe[2], M. Dougal[3], R.H. Elder[1], J.A. Rafferty[1] & G.P. Margison[1]

[1]*CRC Department of Carcinogenesis, Paterson Institute for Cancer Research;* [2]*CRC Department of Medical Oncology and* [3]*Department of Medical Statistics, Christie Hospital NHS Trust, Manchester M20 9BX, UK.*

**Summary** $O^6$-alkylguanine-DNA-alkyltransferase (ATase) levels were measured in extracts of peripheral blood lymphocytes taken at various times during chemotherapy from 19 patients with various haematological malignancies. Seven patients with advanced Hodgkin's disease received preparative treatment consisting of cyclophosphamide (1.5 g $m^{-2}$, daily) administered on days 1 to 4 and BCNU (600 mg $m^{-2}$) on day 5 prior to autologous bone marrow rescue (ABMR) delivered on day 7. Treatment in the remaining 12 patients consisted of cyclophosphamide (1.8 g $m^{-2}$, daily) given on days 1 and 2 followed at day 4 with total body irradiation (TBI) administered in six fractions over the subsequent 3 days to a total dose of 1200 cGy prior to bone marrow transplantation. In the Hodgkin's group, significant decreases in ATase activity were seen during the cyclophosphamide treatment, and the median ATase nadir was 32% (range 0% to 57%) of pretreatment levels following 4 days of cyclophosphamide. In one patient, no ATase activity was detectable following the 4th cyclophosphamide treatment. ATase activities decreased further after BCNU administration to a median of 19% (range 0% to 32%) of pretreatment levels. Extensive cyclophosphamide-induced reduction of lymphocyte ATase levels was also seen in the other group of 12 patients treated with cyclophosphamide/TBI: post-cyclophosphamide median ATase nadir was 35% (range 12% to 78%) of the pretreatment levels. No ATase depletion was seen when cyclophosphamide (up to 10 mM) was incubated for 2 h with pure recombinant human ATase *in vitro* whereas ATase activity was reduced by 90% on preincubation with 100 $\mu$m acrolein or with $>1$ mM phosphoramide mustard. This suggests that a cyclophosphamide-induced decrease in ATase levels in human peripheral lymphocytes *in vivo* may be due to depletion mediated by the production of intracellular acrolein. Since ATase appears to be a principal mechanism in cellular resistance to the cytotoxic effects of BCNU and related alkylating agents, these observations suggest that a cyclophosphamide-induced reduction in ATase activity may be an additional factor in the effectiveness of the combined sequential therapy.

Autologous Bone Marrow Rescue (ABMR) following ablative chemotherapy is being increasingly adopted for patients with high risk advanced Hodgkin's disease, who fail to obtain complete remission following primary induction chemotherapy, relapse within 1 year of completing chemotherapy or who are in second or subsequent relapse after receiving two or more standard chemotherapy regimens (Armitage *et al.*, 1989). Clinical results from recent ABMR series indicate that despite achieving an initial high response rate (range 70–85%), long term cure is achieved in only about 30–35% with most relapse occurring at sites previously involved with disease (Ahmed *et al.*, 1989; Bierman *et al.*, 1988; Carella *et al.*, 1988; Gribben *et al.*, 1989). This indicates that inadequate chemotherapy is the prime reason for failure.

The most popular preparative treatment regimen used for ABMR in Hodgkin's disease involves carmustine (BCNU) in combination with cyclophosphamide and etoposide. This was originally developed by the MD Anderson group (Jagannath *et al.*, 1986; Spitzer *et al.*, 1980) and several variants are currently in use (Ahmed *et al.*, 1989; Bierman *et al.*, 1988; Carella *et al.*, 1988; Gingrich *et al.*, 1990; Gribben *et al.*, 1989; Reece *et al.*, 1991; Teillet *et al.*, 1987). The mechanism of cell kiling by BCNU is initiated by the formation of the mono-adduct, $O^6$-chloroethylguanine which undergoes an intramolecular rearrangement to form $O^6$, N1-ethanoguanine. This then reacts with a cytosine residue in the opposite strand to form a lethal N1-guanine-N3-cytosine ethano DNA cross-link (Brent, 1985; D'Incalci *et al.*, 1988; Gonzaga *et al.*, 1990; Pegg, 1990; Tong *et al.*, 1982). The principal mechanism of BCNU resistance involves the DNA repair enzyme, ATase (Pegg, 1990; D'Incalci *et al.*, 1988) which can remove the chloroethyl group from $O^6$-chloroethylguanine and hence prevent the formation of DNA interstrand cross-links. However, many tumour cells and most tumour cell lines isolated so far have high ATase levels limiting the potential usefulness of BCNU (D'Incalci *et al.*, 1988). One theoretical approach to increasing sensitivity to BCNU is to reduce the levels of ATase prior to administration of the choroethylating agents. This can be achieved in the case of ATase because of its autoinactivating stoichiometric reaction mechanism and its slow rate of resynthesis (Pegg, 1990). Indeed it has been shown in cultured cells that depletion of endogenous ATase by prior exposure to non-toxic doses of monofunctional methylating agents (Futscher *et al.*, 1989; Zlotogorski & Erickson, 1984) or $O^6$-methylguanine (Dolan *et al.*, 1985; Gerson *et al.*, 1988*a*; Yarosh *et al.*, 1986) or $O^6$-benzylguanine (Dolan *et al.*, 1990) rendered the cells more sensitive to subsequent treatment with chloroethylating agents. Conversely, transfer and expression of ATase genes in ATase deficient cells renders them more resistant to chloroethylating agents (Brennand & Margison, 1986; Margison & O'Connor, 1990).

Based on our recent observations of progressive depletion of ATase activity in human peripheral blood lymphocytes of patients with malignant melanoma treated with sequential dacarbazine and fotemustine (Lee *et al.*, 1991*a*), we began to examine the kinetics of ATase depletion following BCNU prior to ABMR. However, results in the first patient to receive sequential cyclophosphamide and BCNU showed an unexpected decrease in ATase activity in the post-cyclophosphamide samples. This observation was pursued and we report here a marked decrease in ATase activity following cyclophosphamide treatment in seven patients with advanced Hodgkin's disease receiving cyclophosphamide and BCNU and in a further group of 12 patients with various haematological malignancies undergoing preparative treatment with cyclophosphamide and total body irradiation (TBI).

Correspondence: S.M. Lee.
Received 27 January 1992; and in revised form 1 April 1992.

### Materials and methods

### *Chemicals*

Cyclophosphamide was obtained from Farmitalia Carlo Erba Ltd; acrolein and glutathione were from Sigma Chemical Co Ltd. and phosphoramide mustard was a generous gift from Dr A. McGown (Paterson Institute, Manchester, UK). Freshly prepared stock solutions (100 mM) in distilled water were used to examine the effects on ATase as described below.

### *Patients and blood samples*

The clinical characteristics of the 19 patients with various haematological malignancies studied are outlined in Table I. All the patients with Hodgkin's disease had failed front line chemotherapy (including MOPP: mechlorethamine, vincristine, procarbazine and prednisolone; ChlVPP: Chlorambucil, vinblastine, procarbazine and prednisolone or HYBRID: Vinblastine, procarbazine, prednisolone, chlorambucil, vincristine, etoposide and adriamycin) and/or salvage chemotherapy (including VAPEC-B: Adramycin, cyclophosphamide, vincristine, bleomycin, etoposide and prednisolone or HYBRID) or had relapsed less than 12 months after chemotherapy. They received preparative treatment consisting of cyclophosphamide (1.5 g $m^{-2}$ i.v., daily) administered on days 1 to 4, BCNU (600 mg $m^{-2}$ i.v.) on day 5 and autologous bone marrow rescue on day 7. The remaining 12 patients (Table I) presented with a variety of haematological diseases and were treated with cyclophosphamide/TBI in which cyclophosphamide (1.8 g $m^{-2}$ i.v., daily) was given on day 1 and 2 followed by six fractionated doses of total body irradiation (200 cGy twice daily) to a total dose of 1,200 cGy from day 4 to 6 prior to allogeneic or autograft marrow transplantation. Patients with acute myeloid leukaemia had previously received combination chemotherapy comprising cytosine arabinoside, daunorubicin and thioguanine and patients with acute lymphoblastic leukaemia had combination chemotherapy with vincristine, daunorubicin, prednisolone, high dose methotrexate followed by intensification with vindesine, asparaginase, cytosine arabinoside, prednisolone and oral maintenance with 6-mercaptopurine and methotrexate. Patients with chronic myeloid leukaemia had previously received hydroxyurea treatment. Serial blood samples were collected at various times during the two preparative regimens. For the cyclophosphamide/BCNU group, blood samples were taken just before chemotherapy and approximately 3, 18, 24, 36, 45, 63, 75, 85, 98, 108, 124 and 132 h after administration of the first dose of cyclophosphamide. For the cyclophosphamide/TBI group, blood samples were taken before and approximately 3, 6, 15, 22, 29, 50,64, 70, 88, 94, 112, 120 h after chemotherapy. Bloods were drawn into a 20 ml universal container containing 0.5 ml of 0.5% EDTA and stored at 4°C before isolation of lymphocytes.

**Table I** Patients characteristics

| Patient | Age/sex | Disease | Treatment | *ATase activity (fm mg⁻¹)* Initial | Nadir |
|---|---|---|---|---|---|
| ST | 17/F | HD IVB | Cyclo/BCNU | 33 | BD[a] |
| SH | 20/M | HD IVB | Cyclo/BCNU | 148 | 47 |
| MR | 50/F | HD IVB | Cyclo/BCNU | 130 | 25 |
| MS | 23/M | HD IVB | Cyclo/BCNU | 170 | 55 |
| RD | 21/M | HD IVB | Cyclo/BCNU | 162 | 12 |
| SJ | 34/M | HD IVB | Cyclo/BCNU | 163 | 53 |
| JC | 39/F | HD IVB | Cyclo/BCNU | 183 | 34 |
| AW | 26/M | AML 1° CR | Cyclo/TBI | 107 | 19 |
| GD | 45/F | AML 1° CR | Cyclo/TBI | 168 | 10 |
| LW | 36/F | AML 1° CR | Cyclo/TBI | 101 | 13 |
| DH | 43/M | AML 1° CR | Cyclo/TBI | 405 | 138 |
| SA | 44/F | ALL 1° CR | Cyclo/TBI | 461 | 95 |
| MC | 18/F | ALL 1° CR | Cyclo/TBI | 148 | 18 |
| HR | 30/F | ALL 1° CR | Cyclo/TBI | 103 | 19 |
| TC | 21/F | ALL 3° CR | Cyclo/TBI | 168 | 37 |
| SK | 28/F | CML 1° CP | Cyclo/TBI | 160 | 23 |
| LB | 38/M | CML 1° CP | Cyclo/TBI | 102 | 45 |
| CN | 38/F | CML 1° CP | Cyclo/TBI | 200 | 18 |
| RB | 54/M | NHL 1° PR | Cyclo/TBI | 107 | 12 |

HD: Hodgkin's disease; AML: acute myeloid leukaemia; ALL: acute lymphoblastic leukaemia; CML: chronic myeloid leukaemia; NHL: non-Hodgkin's lymphoma; CR: complete remission; IVB: stage IVB disease; PR: partial remission; CP: chronic phase; 1°: first; 3°: third. BD[a] = below detection.

### *O⁶-Alkylguanine-DNA alkyltransferase assay*

Lymphocytes (mononuclear cell fraction) were isolated by centrifugation on Ficoll (Pharmacia, Uppsala, Sweden), washed with PBS and centrifuged again into a pellet and stored at $-20$°C. The ATase extraction and assay procedure was carried out as described previously (Lee *et al.*, 1991*a*) with slight modifications. Briefly, cells were disrupted by sonication in 1 ml of buffer I (50 mM Tris–HCl, 3 mM dithiothreitol, 1 mM EDTA, pH 8.3) and centrifuged to prepare cell extracts. Varying amounts of cell extract were incubated with $^{3}$H-methylnitrosourea-methylated calf thymus substrate DNA (specific activity, 19 Ci $mmol^{-1}$) at 37°C for 2 h in a total volume of 500 $\mu$l of 1 mg $ml^{-1}$ bovine serum albumin in buffer I. After incubation, bovine serum albumin (100 $\mu$l of a 10 mg $ml^{-1}$ solution in buffer I) and perchloric acid (200 $\mu$l of a 4 M solution) were added in rapid succession. A further 2 ml of 1 M perchloric acid was added and the mixture heated at 75°C for 45 min. Samples were clarified by centrifugation and the precipitates were washed with 4 ml of 1 M perchloric acid before being resuspended in 300 $\mu$l of 0.01 M sodium hydroxide and dissolved in 3 ml of aqueous scintillation fluid (Ecoscint A; National Diagnostics). Counting efficiency was approximately 28%. Specific activity measurements were based on a minimum of three points on the linear part of the curve. ATase activity was expressed as fmoles methyl transferred to protein per mg of total protein in the extract, protein concentration being measured using the Bradford method with bovine serum albumin as the standard (Bradford, 1976). Statistical analysis was based on repeated measurement analysis using the BMDP statistical software program and was done on the pre-chemotherapy and pre-TBI assays.

In order to assess the ability of cyclophosphamide, acrolein and phosphoramide mustard to inhibit the ATase *in vitro*, varying concentrations of these agents were incubated with 70 fmoles of pure recombinant human ATase (Santibanez-Koref *et al.*, 1992 in press) for 2 h at 37°C in buffer I without dithiothreitol. Residual ATase activity was then measured by incubation with excess substrate DNA. The effect of glutathione on the inhibition of ATase by acrolein was monitored by incubating various amounts of gluthathione for 2 h with 500$\mu$M acrolein, a concentration that caused a 95% depletion in ATase activity *in vitro*. Following this, 70 fmoles of recombinant ATase was added and the experiment continued as above.

### Results

### *Decrease in ATase* in vivo *following cyclophosphamide and BCNU*

Pretreatment ATase levels in the seven Hodgkin's patients ranged from 33 to 183 (mean 141) fm $mg^{-1}$ total protein. In all seven patients, decreases in ATase activity were seen following cyclophosphamide administration. Wide variations were noted in the rates and extents of ATase reduction between various individuals (see Figures 1a and 1b). In three patients the first cyclophosphamide treatment caused reduction to 52%, 64% and 67% of their pretreatment levels (patients RD, SH and ST, Figures 1a and 1b) while in two other patients the first treatment caused only a 10% loss in activity (patients SJ and JC, Figure 1b). Following four cyclophosphamide treatments the median ATase nadir was 32% (range 0% to 57%) of pretreatment levels. In one patient (ST,

**Figure 1** O[6]-alkylguanine-DNA-alkyltransferase (ATase) specific activity (fmoles $mg^{-1}$ protein) in extracts of peripheral lymphocytes of Hodgkin's patients sampled at various times after high dose cyclophosphamide given on day 1, 2, 3 and 4 and BCNU given on day 5. **a**, Patients ST (△), SH (▲), MR (○) and MS (●). **b**, Patients RD (△), SJ (▲) and JC (○).

**Figure 3** Consensus data for O[6]-alkylguanine-DNA-alkyltransferase (ATase) specific activity (fmoles $mg^{-1}$ protein) in extracts of peripheral lymphocytes of **a**, patients treated with cyclophosphamide and BCNU (see Figure 1) and **b**, patients treated with cyclophosphamide and TBI (see Figure 2). Figures show mean values (●) and upper (▼) and lower (▲) 95% confidence intervals.

Figure 1a), no ATase activity was detectable after the fourth cyclophosphamide treatment. In two patients there was evidence of partial regeneration of ATase during the cyclophosphamide treatments (SH and SJ, Figures 1a and 1b). ATase activities continued to decrease after BCNU administration to a median of 19% (range 0% to 32%) of pretreatment levels.

Although in some case, the loss of ATase was small after administration of BCNU; overall there was a substantial post-BCNU decrease in ATase and this was highly statistically significant: maximal loss occurred between the second and fourth dose of cyclophosphamide ($P = 0.0013$) and after BCNU administration ($P = 0.0018$). A consensus summary of the data from all seven patients is shown in Figure 3a.

### *Decrease in ATase* in vivo *following cyclophosphamide and TBI*

Pretreatment ATase levels in the 12 patients monitored ranged from 101 to 462 (mean 186) fm $mg^{-1}$. As above, the extents and rates of decrease in activity were variable with a median post-cyclophosphamide nadir of 35% (range 12% to 78%) of pretreatment levels. The extent of ATase loss did not appear to be related to the pretreatment levels. In seven patients there was a transient increase in ATase activity after the first dose of cyclophosphamide (SA, MR, HR, TC, LB, RB and CN, Figures 2b and 2c) followed by reduction to nadirs from 80% to 10% of pre-cyclophosphamide levels. In two patients significant partial recovery of ATase was seen during the cyclophosphamide treatments and prior to TBI (RB and SK, Figure 2c). Generally, relatively small changes in ATase activity were seen during the TBI treatments. However, in three patients TBI itself appeared to extensively suppress ATase activity (RB, TC and CN, Figures 2b and 2c) but in two of these (TC and CN, Figures 2b and 2c) an initial marked suppression was followed by an equally extensive recovery.

Statistical analysis again showed that the effect of cyclophosphamide on ATase was cumulative, particularly between the first dose of cyclophosphamide and the second day of TBI ($P < 0.0001$). A consensus summary of the data from all 12 patients is shown in Figure 3b. Although the mean pretreatment ATase activity was higher and the ATase reduction rate appears faster than in the Hodgkin's group, this was not statistically significant ($P = 0.097$ and $P = 0.89$ respectively) and there was therefore no correlation between pretreatment history and ATase loss. The apparently greater individual variation in ATase in the cyclophosphamide/TBI than the cyclophosphamide/BCNU group might be related to the fact that the former group received two and the latter four consecutive days of cyclophosphamide treatment, or to the different pretreatment history.

There was progressive reduction in WBC counts over the 5–7 days of cyclophosphamide/BCNU and cyclophospha-

**Figure 2** O[6]-alkylguanine-DNA-alkyltransferase (ATase) specific activity (fmoles $mg^{-1}$ protein) in extracts of peripheral lymphocytes of patients sampled at various times after cyclophosphamide given on day 1 and 2 and TBI given on days 4, 5 and 6. **a**, Patients AW (▲), GD (△), CW (○) and DH (●). **b**, Patients SA (▲), MR (△), HR (○) and TC (●). **c**, Patients SK (▲), LB (△), CN (○) and RB (●).

**Figure 4** **a**, Effect of pre-incubation of pure recombinant human $O^6$-alkylguanine-DNA-alkyltransferase (ATase) with increasing concentrations of cyclophosphamide (●), phosphoramide mustard (○) or acrolein (□). See text for experimental details. **b** Effect of preincubation of acrolein with increasing concentrations of glutathione (GSH) on its ability to deplete the activity of pure recombinant human ATase.

mide/TBI: the mean pretreatment leucocyte count was $7.04 \times 10^9$ $l^{-1}$ and mean post-cyclophosphamide leucocyte count was $3.18 \times 10^9 l^{-1}$.

### *ATase depletion* in vitro *following incubation with cyclophosphamide and its metabolites*

The direct effects of cyclophosphamide, acrolein and phosphoramide mustard on ATase were also assessed by incubating the drugs with a fixed amount of pure recombinant human ATase for 2 h at 37°C *in vitro*. Figure 4a shows the dose-response curves for ATase depletion following incubation with the above drugs. No ATase depletion was seen when recombinant human ATase was incubated with cyclophosphamide. By contrast, acrolein was a highly effective inactivator of the enzyme, in that under the conditions used, only 100 μM caused 90% depletion. ATase depletion was also seen with phosphoramide mustard but this was with a concentration far in excess of that achievable in patients receiving the drug (>1 mM) (Jardine *et al.*, 1978; Sladek *et al.*, 1984; Juma *et al.*, 1979).

Increasing concentrations of glutathione were also incubated with 500 μM acrolein which caused 95% depletion of ATase activity in the competition assay above. As shown in Figure 4b, 1 mM glutathione was able to completely prevent acrolein-induced depletion of ATase.

### Discussion

In the present study we have shown extensive decreases in ATase activity in peripheral blood lymphocytes of 19 patients receiving cyclophosphamide preparative treatment prior to bone marrow transplantation. Wide interindividual variations in the pretreatment levels and in the rate of ATase loss was noted. In the Hodgkin's patients the effect was so marked that after the 4th cyclophosphamide treatment the median ATase nadir was 32% (range 0 to 57%). In one patient (Figure 1a) no ATase activity was detected following the 4th cyclophosphamide administration. The two patients that showed least overall decrease immediately prior to BCNU demonstrated partial recovery of ATase activity during the cyclophosphamide treatments and this may have contributed to the overall lower ATase reduction (Figures 1a and 1b). Although in some cases, the loss of ATase was minor after administration of BCNU, overall there was a substantial decrease in ATase and this was highly statistically significant with maximal loss occuring between the second and fourth dose of cyclophosphamide ($P = 0.0013$) and after BCNU administration ($P = 0.0018$) (Figure 3a). The significant reduction of ATase observed, agrees with that of another study (Gerson, 1989) using 350 mg $m^{-2}$ of BCNU.

A similar picture emerged in the other group of 12 patients treated with cyclophosphamide/TBI, with a median post-cyclophosphamide nadir of 39%. In two patients partial recovery of ATase was seen during the cyclophosphamide treatments and prior to TBI (Figures 2b and 2c). In two patients there was some indication that TBI itself was associated with a transient (Figures 2b and 2c), and in one patient, continued (Figure 2c) suppression of ATase activity. Clearly this effect requires substantiation with a large number of patients receiving only TBI. It is interesting to note that in rodents, ATase activity in a number of tissues was increased by a single dose of ionising radiation (Margison *et al.*, 1985; Stammberger *et al.*, 1990).

Changes in the specific activity of peripheral lymphocyte ATase might be the consequence of cyclophosphamide-induced changes in the lymphocyte population: transient increases in ATase activity were seen in some patients but the changes were slight and may have been due to experimental variation or intra-individual variation. However, in the latter case it has been shown that most individuals have characteristic lymphocyte ATase levels over a short term period and this is therefore unlikely to contribute to the overall picture (Gerson *et al.*, 1985; Sagher *et al.*, 1988). It is also possible that clonal selection may have occurred as a consequence of cytolysis of a lymphocyte population(s) with relatively low ATase specific activity. The possibility that continued cytolysis may have contributed to the consistent decrease in ATase by affecting those lymphocytes with the highest specific activity cannot be excluded since white cell counts had decreased by approximately 50% post cyclophosphamide. Whilst the relative contribution of T and B lymphocytes to the overall ATase measurements was not assessed in this study, previous reports have shown ATase specific activities of 190 and 140 fm $mg^{-1}$ respectively (Gerson *et al.*, 1985). However, the overall changes we have observed are unlikely to be attributable to T or B specific effects since B lymphocytes make up only a small proportion of the total population and the proportion of T and B cells is similar in Hodgkin's, non-Hodgkin's lymphoma and controls (Herrmann *et al.*, 1983).

Another possible explanation for ATase loss is that there is a direct depleting effect on the ATase itself: as far as we are aware, $O^6$-alkylguanine lesions have not yet been identified in DNA *in vivo* after administration of cyclophosphamide or its metabolites. There are two reports (Kleihues & Margison, 1976; Meer *et al.*, 1989) which showed that cyclophosphamide is able to increase the amount of $O^6$-methylguanine in DNA following a chasing dose of methylating agent in rodent liver and both authors attributed this to some as yet unidentified $O^6$-alkylation product of guanine in DNA which is repaired by ATase and results in ATase depletion. Alternatively, there may be a direct reaction of the cyclophosphamide metabolite acrolein with ATase: when given systemically, cyclophosphamide is metabolised by the hepatic mixed-function oxidases, to 4-hydroxycyclophosphamide, the 'transport' form which enters cells and eventually decomposes intracellularly to phosphoramide mustard, the ultimate cross-linking metabolite of cyclophosphamide, and acrolein (Brock, 1989; Sladek, 1987). We have shown that 100 μM acrolein is able to deplete ATase activity when it was incubated *in vitro* with pure recombinant human ATase. This may be the result of the affinity of acrolein for sulfhydryl groups including, possibly, the alkyl-accepting cysteine residue of the ATase protein. The peak concentration of phosphoramide mustard achieved in the serum following high dose cyclophosphamide (60 and 75 mg $kg^{-1}$) was 50–100 μM (Colvin & Chabner, 1990; Jardine *et al.*, 1978; Juma *et al.*, 1979) indicating that the concentration of intracellular acrolein that depletes recombinant human ATase *in vitro* is potentially attainable *in vivo*. Phosphoramide mustard was also able to deplete ATase activity but the concentration required (> 1 mM) was far in excess of that achievable in patients receiving the drug (Colvin & Chabner, 1990; Jardine *et al.*, 1978; Sladek *et al.*, 1984).

The variation in ATase decreases seen in the 19 patients studies following cyclophosphamide treatment may be due to the differential metabolism of cyclophosphamide or related to variations in cellular glutathione and glutathione transferase levels in different individuals, as both are responsible for the intracellular metabolism and detoxification of various cyclophosphamide metabolites (Chresta *et al.*, 1990; Draeger *et al.*, 1976; Lee, 1991*b*; McGown & Fox, 1986). It has previously been demonstrated that the amount of the ultimate active metabolites formed intracellularly is dependent on the intracellular glutathione concentration and its interaction with the toxic metabolites (Lee *et al.*, 1991*c*). Cyclophosphamide has also been shown to be able to deplete serum glutathione (Carmichael *et al.*, 1986). Here we were able to show that glutathione can inhibit acrolein-induced depletion of ATase, supporting the hypothesis that in the case of lymphocytes, intracellular glutathione levels may be one of the factors that determines the extent of ATase decrease.

Irrespective of the mechanism, our observations on peripheral lymphocytes may have important general implications in combination chemotherapy if similar changes in ATase occur in the tumour and indeed, if the extents of ATase loss achieved are sufficient to sensitise the tumour cells to killing by agents such as BCNU. In cultured human tumour cells lines that express high levels of ATase, depletion of the enzyme following exposure to $O^6$-benzylguanine increases their sensitivity to the toxic effects of subsequent doses of chloroethylating agents, greater extents of sensitisation being produced in cells expressing higher levels of ATase: no such sensitisation occurs in equivalent ATase deficient cells (Dolan *et al.*, 1991). It might therefore be argued that enzyme depletion in ATase-expressing tumour cells would considerably enhance chemotherapeutic effectiveness without significantly increasing the toxic side effects in tissues such as bone marrow that generally express low levels of the enzyme.

It is not unreasonable to suggest that the ATase-reducing action of cyclophosphamide would best be exploited by employing sequential cyclophosphamide and BCNU (as here) rather than a regimen in which cyclophosphamide and BCNU are administered concurrently or in which BCNU is given before cyclophosphamide. Further support for such a schedule is that intracellularly released acrolein has been shown to deplete cellular glutathione (Gurtoo *et al.*, 1981; Lee, 1991*b*) and the latter is able to decrease the cytotoxic and DNA cross-linking activity of BCNU (Ali-Osman *et al.*, 1989). Therefore, if used as in the schedule described here, the glutathione-depleting property of acrolein may further sensitise tumour cells to BCNU. In support of this suggestion it is interesting to note that in one series of 54 patients with advanced Hodgkin's disease undergoing ABMR (Reece *et al.*, 1991), where the condition schedule involved administering BCNU after cyclophosphamide together with etoposide, the complete response rate and disease-free survival rate were 80% and 55% respectively. In contrast, most other series using similar preparative drugs, but different schedules, the complete response rate averaged about 45% and only approximately 10 to 30% achieved disease-free survival (Ahmed *et al.*, 1989; Bierman *et al.*, 1988; Carella *et al.*, 1988; Jagannath *et al.*, 1986).

In conclusion, cyclophosphamide is capable of reducing ATase activity in peripheral lymphocytes and one possible explanation is that this is mediated via the release of intracellular acrolein, a cyclophosphamide metabolite. This property could be exploited in designing future combination chemotherapy schedules; this method of reducing cellular ATase levels may be an alternative to the proposed use of agents such as $O^6$-benzylguanine since, in the case of cyclophosphamide, reduction is accomplished by an agent with proven antitumour activity.

We are grateful to the nursing staff of Adult Leukaemic Unit, Christie Hospital for blood sampling. This work was supported by funds from the Cancer Research Campaign, UK.

### References

AHMED, T., CIAVARELLA, D., FELDMAN, E., ASCENSAO, J., HUSSAIN, F., ENGELKING, C., GINGRICH, S., MITTELMAN, A., COLEMAN, M. & ARLIN, Z.A. (1989). High-dose potentially myeloablative chemotherapy and autologous bone marrow transplantation for patients with advanced Hodgkin's disease. *Leukemia*, **3**, 19–32.

ALI-OSMAN, F., CAUGHLAN, J. & GRAY, G.S. (1989). Decreased DNA interstrand cross-linking and cytotoxicity induced in human brain tumour cells by 1,3-bis(2-chloroethyl)-1-nitrosourea after *in vitro* reaction with glutathione. *Cancer Res.*, **49**, 5954–5948.

ARMITAGE, J.O., BARNETT, M.J., CARELLA, A.M., DICKE, K.A., DIEHL, V., GRIBBEN, J.G. & PREUNDSCHUH, M. (1989). Bone marrow transplantation in the treatment of Hodgkin's lymphoma: problems, remaining challenges and future prospects. In *New Aspects in Diagnosis and Treatment of Hodgkin's Disease*. Diehl, V., Pfreundschuh, M. & Loeffler, M. (eds), pp. 246–253. Springer-Verlag: Berlin-Heidelberg.

BIERMAN, P.J., JAGANNATH, S., DICKE, K.A., KESSINGER, A., HAGERRMEISTER, F.B., VOSE, J.M., HORWITZ, L.J., CABANILLAS, F., VAUGHAN, W.P., SPITZER, G. & ARMITAGE, J.O. (1988). High dose cyclophosphamide, carmustine and etoposide (CBV) in 128 patients with Hodgkin's disease. *Blood*, **72** (suppl 1), 239a.

BRADFORD, M.M. (1976). A rapid and sensitive method for the quantitation of microgram quantities of protein utilising the principle of protein-dye binding. *Anal. Biochem.*, **72**, 248–254.

BRENNAD, J. & MARGISON, G.P. (1986). Reduction of the toxicity and mutagenicity of alkylating agents in mammalian cells harboring the *Escherichia coli* alkyltransferase gene. *Proc. Natl Acad. Sci. USA*, **83**, 6292–6296.

BRENT, T.P. (1985). Isolation and purification of $O^6$-alkylguanine-DNA alkyltransferase from human leukemic cells: prevention of chloroethylnitrosourea-induced cross-links by purified enzyme. *Pharmacol. Ther.*, **31**, 121–140.

BROCK, N. (1989). Oxazaphosphorine cytostatics: past-present-future. Seventh Cain Memorial Award Lecture. *Cancer Res.*, **49**, 1–7.

CARELLA, A., CONGIU, A.M., GAOZZA, E., MAZZA, P., RICCI, P., VISANI, G., MELANI, G., CIMINO, G., MANGONI, L., COSER, P., CETTO, G.L., CIMINO, R., ALESSANDRINO, E.P., BRUSAMOLINO, E., SANTINI, G., TURA, S., MANDELLI, F., RIZZOLI, V., BERNASCONI, C. & MARMONT, A.M. (1988). High-dose chemotherapy with autologous bone marrow transplantation in 50 advanced resistant Hodgkin's disease patients: an Italian group report. *J. Clin. Oncol.*, **6**, 1411–1416.

CARMICHAEL, J., ADAMS, D.J., ANSELL, J. & WOLF, R. (1986). Glutathione and glutathione transferase levels in mouse granulocytes following cyclophosphamide administration. *Cancer Res.*, **46**, 735–739.

CHRESTA, C.M., CROOK, T.R. & SOUHAMI, R.L. (1990). Depletion of cellular glutathione by N,N′-Bis (*trans*-4-hydrocyclohexyl)-N′-nitrosourea as a determinant of sensitivity of K562 human leukemia cells to 4-hydroperoxycyclophosphamide. *Cancer Res.*, **50**, 4067.

COLVIN, M. & CHABNER, B.A. (1990). Alkylating agents. In *Cancer Chemotherapy: Principles and Practice*, Chabner, B.A. & Collins, J.M. (eds), pp. 276–313. Lippincott: Philadelphia.

D'INCALCI, M., CITTI, L., TAVERNA, P. & CATAPANO, C.V. (1988). Importance of DNA repair enzyme $O^6$-alkyltransferase (AT) in cancer chemotherapy. *Cancer Treat. Rev.*, **15**, 279–292.

DOLAN, M.E., CORSICO, C.D. & PEGG, A.E. (1985). Exposure of HeLa cells to O[6]-alkylguanines increases sensitivity to the cytotoxic effects of alkylating agents. *Biochem. Biophys. Res. Commun.*, **132,** 178–185.

DOLAN, M.E., ROBERT, C.M. & PEGG, A.E. (1990). Depletion of mammalian O[6]-alkylguanine-DNA alkyltransferase activity by O[6]-benzylguanine provides a means to evaluate the role of this protein in protection against carcinogenic and therapeutic agents. *Proc. Natl Acad. Sci. USA*, **87,** 5368–5372.

DOLAN, M.E., MITCHELL, R.B., MUMMERT, C., MOSCHEL, R.C. & PEGG, A.E. (1991). Effects of O[6]-benzylguanine analogues on sensitivity of human tumor cells to the cytotoxic effects of alkylating agents. *Cancer Res.*, **51,** 3367–3372.

DRAEGER, U., PETER, G. & HOHORST, H.J. (1976). Deactivation of cyclophosphamide (NSC-26271) metabolites by sulphydryl compounds. *Cancer Treat. Rep.*, **60,** 355–359.

FUTSCHER, B.W., MICETICH, K.C., BARNES, D.M., FISHER, R.I. & ERICKSON, L.C. (1989). Inhibition of a specific DNA repair system and nitrosourea cytotoxicity in resistant human cancer cells. *Cancer Commun.*, **1,** 65–73.

GERSON, S.L., MILLER, K. & BERGER, N.A. (1985). O[6]-alkylguanine-DNA alkyltransferase activity in myeloid cells. *J. Clin. Invest.*, **76,** 2106–2114.

GERSON, S.L., TREY, J.E. & MILLER, K. (1988). Potentiation of nitrosourea cytotoxicity in human leukemic cells by inactivation of O[6]-alkylguanine-DNA alkyltransferase. *Cancer Res.*, **48,** 1521–1527.

GERSON, S.L. (1989). Modulation of human lymphocyte O[6]-alkylguanine- DNA alkyltransferase by streptozotocin *in vivo*. *Cancer Res.*, **49,** 3134–3138.

GINGRICH, R.D., GINDER, G.D., BURNS, L.J., WEN, B.-C. & FYFE, M.A. (1990). BVAC ablative chemotherapy followed by autologous bone marrow transplantation for patients with advanced lymphoma. *Blood*, **75,** 2276–2281.

GONZAGA, P.E., HARRIS, L., MARGISON, G.P. & BRENT, T.P. (1990). Evidence that covalent complex formation between BCNU-treated oligonucleotides and *E. coli* alkyltransferases requires the O6-alkylguanine function. *Nucleic Acids Res.*, **18,** 3961–3966.

GRIBBEN, J.G., LINCH, D.C., SINGER, C.R.J., MCMILLAN, A.K., JARRETT, M. & GOLDSTONE, A.H. (1989). Successful treatment of refractory Hodgkin's disease by high-dose combination chemotherapy and autologous bone marrow transplantation. *Blood*, **73,** 340–344.

GURTOO, H.L., HIPKENS, J.H. & SHARMA, S.D. (1981). Role of glutathione in the metabolism-dependent toxicity and chemotherapy of cyclophosphamide. *Cancer Res.*, **41,** 3584–3591.

HERRMANN, F., SIEBER, G., JAUER, B., LOCHNER, A., KOMISCHKE, B. & RUHL, H. (1983). Evaluation of the circulating and splenic lymphocyte subpopulations in patients with non-Hodgkin's lymphomas and Hodgkin's disease using monoclonal antibodies. *Blut*, **47,** 41–51.

JAGANNATH, S., DICKE, K.A., ARMITAGE, J.O., CABANILLAS, F., HORWITZ, L.J., VELLEKOOP, L., ZANDER, A.R. & SPITZER, G. (1986). High dose cyclophosphamide, carmustine and etoposide and autologous bone marrow transplantation for relapsed Hodgkin's disease. *Ann. Intern. Med.*, **1024,** 4163–4168.

JARDINE, I., FENSELAU, C., APPLER, M., KAN, M-N., BRUNDRETT, R.B. & COLVIN, M. (1978). Quantitation by gas chromatography-chemical ionization mass spectrometry of cyclophosphamide, phosphoramide mustard, and nornitrogen mustard in the plasma and urine of patients receiving cyclophosphamide therapy. *Cancer Res.*, **38,** 408–415.

JUMA, F.D., ROGERS, H.J. & TROUNCE, J.R. (1979). The pharmacokinetics of cyclophosphamide, phosphoramide mustard and nornitrogen mustard studied by gas chromatography in patients receiving cyclophosphamide therapy. *Br. J. Clin. Pharmacol.*, **10,** 209–217.

KLEIHUES, P. & MARGISON, G.P. (1976). Exhausion and recovery of repair excision of O[6]-methylguanine from rat liver DNA. *Nature*, **259,** 153–155.

LEE, S.M., THATCHER, N. & MARGISON, G.P. (1991*a*). O[6]-alkylguanine-DNA alkyltransferase depletion and regeneration in human peripheral lymphocytes following dacarbazine and fotemustine. *Cancer Res.*, **51,** 619–623.

LEE, F.Y.F. (1991*b*). Glutathione diminishes the anti-tumour activity of 4-hydroperoxycyclophosphamide by stabilising its spontaneous breakdown to alylating metabolites. *Br. J. Cancer*, **63,** 45–50.

LEE, F.Y.F., FLANNERY, D.J. & SIEMANN, D.W. (1991*c*). Prediction of tumour sensitivity to 4-hydroperoxycyclophosphamide by a glutathione-targeted assay. *Br. J. Cancer*, **63,** 217–222.

MARGISON, G.P., BUTLER, J. & HOEY, B. (1985). O[6]-methylguanine activity is increased in rat tissues by ionising radiation. *Carcinogenesis*, **6,** 1699–1702.

MARGISON, G.P. & O'CONNOR, P.J. (1990). Biological consequences of reactions with DNA: role of specific lesions. In *Handbook of Experimental Pharmacology, Vol 94/I.* Cooper, C.S. & Grover, P.L. (eds), pp. 547–571. Springer-Verlag: Berlin-Heidelberg.

MCGOWN, A.T. & FOX, B.W. (1986). A proposed mechanism of resistance to cyclophosphamide and phosphoramide mustard in a Yoshida cell line *in vitro*. *Cancer Chemother. Pharmacol.*, **17,** 223–226.

MEER, L., SCHOLD, S.C. & KLEIHUES, P. (1989). Inhibition of the hepatic O[6]-alkylguanine-DNA alkyltransferase *in vivo* by pretreatment with antineoplastic agents. *Biochem. Pharmacol.*, **38,** 929–934.

PEGG, A.E. (1990). Mammalian O[6]-alkylguanine-DNA alkyltransferase: regulation and importance in repsonse to alkylating carcinogenic and therapeutic agents. *Cancer Res.*, **50,** 6119–6129.

REECE, D.E., BARNETT, M.J., CONNORS, J.M., FAIREY, R.N., GREER, J.P., HERZIG, G.P., HERZIG, R.H., KLINGEMANN, H-G., O'REILLY, S.E., SHEPHERD, J.D., SPINELLI, J.J., VOSS, N.J., WOLFF, S.N. & PHILLIPS, G.L. (1991). Intensive chemotherapy with cyclophosphamide, carmustine, and etoposide followed by autologous bone marrow transplantation for relapsed Hodgkin's disease. *J. Clin. Oncol.*, **9,** 1871–1879.

SAGHER, D., KARRISON, T., SCHWARTZ, J.L., LARSON, R., MEIER, P. & STRAUSS, B. (1988). Low O[6]-alkylguanine DNA alkyltransferase activity in the peripheral blood lymphocytes of patients with therapy-related acute nonlymphocytic leukemia. *Cancer Res.*, **48,** 3084–3089.

SANTIBANEZ-KOREF, M., ELDER, R.H., FAN, C.-Y., CAWKWELL, L., MCKIE, J.H., DOUGLAS, K.T., MARGISON, G.P. & RAFFERTY, J.A. (1992). Isolation and partial characterisation of murine O[6]-alkylguanine-DNA-alkyltransferase; comparative sequence and structural properties. *Molecular Carcinogenesis*, **5,** 161–169.

SLADEK, N.E., DOEDEN, D., POWERS, J.F. & KRIVIT, W. (1984). Plasma concentrations of 4-hydroxycyclopohosphamide and phosphoramide mustard in patients repeatedly given high doses of cyclophosphamide in preparation for bone marrow transplantation. *Cancer Treat. Rep.*, **68,** 1247–1254.

SLADEK, N.E. (1987). Oxazaphosphorines. In *Metabolism and Action of Anti-Cancer Drugs*, Powis, G. & Prough, R.A. (eds), pp. 48–90. Taylor and Francis: London.

SPITZER, G., DICKE, K.A., LITAM, J., VERMA, D.S., ZANDER, A., LANZOTTI, V., VALDIVIESO, M., MCCREDIE, K.B. & SAMUELS, M.L. (1980). High dose combination chemotherapy with autologous bone marrow transplantation in adult solid tumours. *Cancer*, **45,** 3075–3085.

STAMMBERGER, I., SCHMAHL, W. & NICE, L. (1990). The effects of x-irradiation, N-ethyl-N-nitrosourea or combined treatment on O[6]-alkylguanine-DNA alkyltransferase activity in fetal rat brain and liver and the induction of CNS tumours. *Carcinogenesis*, **11,** 219–222.

TEILLET, F., PULIK, M., TEILLET-THIEBAND, F., BLAISE, A.M., KUENTZ, M., COURTOIS, F., ANDOLENKO, P., BLEICHNER, G. & COSTE, F. (1987). Autologous bone marrow transplantation (ABMT) in poor prognosis Hodgkin's disease. *Bone Marrow Transplant*, **2** (suppl 1), 211.

TONG, W.P., KIRK, M.C. & LUDLUM, D.B. (1982). Formation of the crosslink 1-[N[3]-deoxycytidyl],2-[N[1]-doxyguanosinyl]ethane in DNA treated with N,N-bis(chloroethyl)-N-nitrosourea. *Cancer Res.*, **42,** 3102–3105.

YAROSH, D.B., HURST-CALDERONE, S., BABICH, M.A. & DAY, R.S. III (1986). Inactivation of O[6]-methylguanine-DNA methyltransferase and sensitization of human tumour cells to killing by chloroethylnitrosourea by O[6]-methylguanine as a free base. *Cancer Res.*, **46,** 1663–1668.

ZLOTOGORSKI, C. & ERICKSON, L.C. (1984). Pretreatment of human colon tumour cells with DNA methylating agents inhibits their ability to repair chloroethyl monoadducts. *Carcinogenesis*, **5,** 83–87.